# Progressive Liver Disease in Patients With Ataxia Telangiectasia

**DOI:** 10.3389/fped.2019.00458

**Published:** 2019-11-07

**Authors:** Helena Donath, Sandra Woelke, Marius Theis, Ursula Heß, Viola Knop, Eva Herrmann, Dorothea Krauskopf, Matthias Kieslich, Ralf Schubert, Stefan Zielen

**Affiliations:** ^1^Division of Allergology, Department for Children and Adolescents, Pulmonology and Cystic Fibrosis, Goethe University, Frankfurt, Germany; ^2^Division of Pediatric Neurology, Department for Children and Adolescents, Goethe University, Frankfurt, Germany; ^3^Department for Internal Medicine, Goethe University, Frankfurt, Germany; ^4^Institute of Biostatistics and Mathematical Modeling, Goethe University, Frankfurt, Germany

**Keywords:** Ataxia telangiectasia, liver disease, hepatic steatosis, neurodegeneration, ataxia score

## Abstract

Ataxia telangiectasia (A-T) is a devastating multi-system disorder characterized by progressive cerebellar ataxia, immunodeficiency, genetic instability, premature aging and growth retardation. Due to better care the patients get older than in the past and new disease entities like disturbed glucose tolerance and liver disease emerge. The objective of the present investigation is to determine the evolution of liver disease and its relation to age and neurological deterioration. The study included 67 patients aged 1 to 38 years with classical A-T. At least two measurements of liver enzymes were performed within a minimum interval of 6 months in 56 patients. The median follow-up period was 4 years (1–16 years). A total of 316 liver enzyme measurements were performed. For analysis, patients were divided into two age groups (Group 1: <12 years; group 2: ≥12 years). In addition, ultrasound of the liver and Klockgether Ataxia Score (KAS) were analyzed. We found significantly higher levels of alpha-fetoprotein (AFP) (226,8 ± 20.87 ng/ml vs. 565,1 ± 24.3 ng/ml, *p* < 0.0001), and liver enzymes like ALT (23.52 ± 0.77 IU/L vs. 87.83 ± 5.31 IU/L, *p* < 0.0001) in patients in group 2. In addition, we could show a significant correlation between age and AFP, GGT, and KAS. Ultrasound revealed hepatic steatosis in 11/19 (57.9%) patients in group 2. One female patient aged 37 years died due to a hepato-cellular carcinoma (HCC). Liver disease is present in the majority of older A-T patients. Structural changes, non-alcoholic fatty liver disease and fibrosis are frequent findings. Progress of liver disease is concomitant to neurological deterioration.

## Introduction

Ataxia telangiectasia (A-T) is a rare devastating human autosomal recessive disorder characterized by progressive cerebellar ataxia, immunodeficiency, growth retardation, chromosomal instability, and cancer susceptibility (1–3). Recurrent respiratory infections and cancer are the major reasons for death in A-T (4, 5). Due to better care, the majority of patients get older than in the past and new disease entities like disturbed glucose tolerance and liver disease emerge (6). Disease progression of A-T is evident at different organ systems. Neurological decline, progressive lung disease and, recently, metabolic alterations, and liver disease have been described as prominent features (6, 7). Still, little is known about the natural course of hepatopathy in A-T. Liver enzymes such as gamma-glutamyl-transferase (GGT), aspartate aminotransferase (AST), and alanine aminotransferase (ALT) are elevated in older patients, suggesting hepatic dysfunction (8). In addition, high levels of alpha-fetoprotein (AFP) are characteristic findings among affected patients at all ages. Normally, AFP production in the fetal liver ceases after embryonal differentiation until the age of one (9), whereas in A-T it is slowly increasing with age (10). Remarkably, post-mortem analyses of the liver in A-T patients showed severe pathological findings like hepatitis with periportal fibrosis (11) or even cirrhosis (12). There are few clinical case reports on histological finding of non-alcoholic steatosis hepatis (NASH), liver cirrhosis and hepato-cellular carcinoma (HCC) in A-T (7, 13, 14). The first cohort study of prevalence and nature of hepatic involvement in patients with A-T was published recently by Weiss et al. (7). The authors reported that abnormal liver enzymes were present in 43.4% of A-T patients. In addition, dyslipidemia with increased total cholesterol and triglycerides was detected in some older patients (6, 7). A recent study from Paulino et al. (15) in 17 A-T patients reported that diabetes and liver disease is demonstrable by atherosclerotic lipid profiling; in addition they found hepatic steatosis in 64.7% of their patients which tends to get worse as they become older. Some of the patients suffer from metformin-resistant diabetes, which typically occurs from adolescence on (2, 6). However, reduced insulin sensitivity and dysglycemia can also be observed in A-T patients without evident diabetes (15, 16). There is growing evidence for a correlation between dyslipidemia, insulin resistance and the development of a fatty liver disease (7, 15). Still, the search for a possible etiology of liver disease (virus serology, autoantibodies, iron, copper metabolism, alpha-1-antitrypsin, C-reactive protein (CRP), thyroid function test) in A-T did not show significant findings (7). Thus, liver disease progression may be caused intrinsically by multiple factors of which inflammation and oxidative stress may be of particular importance (17–20).

Apart from these investigations, literature is still scarce on liver involvement in A-T. Although not investigated in previous studies, it can be speculated that liver disease indicates a more severe course of A-T, going along or maybe even aggravating neurological decline. In general, liver disease leads to accumulation of toxic metabolites, e.g., ammoniac, which harms the nervous system. It is well known for decades that there is a relationship between normal liver cell function and brain (21–23). Pruritus in cholestasis patients and profound fatigue are often described in chronic liver disease and both symptoms are commonly reported by older A-T patients (1, 2).Nevertheless, the relationship between fatigue, pruritus and liver disease is unclear and may be in part due to premature aging of the skin and not caused by liver disease.

In this investigation we aimed to characterize not only the evolution of liver disease by liver enzymes, CRP, abdominal ultrasound but also its relation to the neurological status studied by Klockgether Ataxia Score (KAS) in a large cohort of A-T patients.

## Materials and Methods

In a retrospective analysis from August 2002 to August 2018 we studied data of 67 classical A-T patients from the Frankfurt A-T cohort aged 1 to 38 years regarding CRP, liver enzymes (GGT, ALT, AST), abdominal ultrasound and neurological status according to the KAS (24). Longitudinal data were available of 56 patients. All patients were clinically and/or genetically diagnosed with A-T according to recent World Health Organization (WHO) recommendations.

We compared patients <12 years of age (group 1, *n* = 37) to patient ≥12 years (group 2, *n* = 30). Patients with longitudinal data in both groups were assigned to the group for which more measurements were available.

Patients who had experienced chemotherapy and/or stem cell transplantation (*n* = 5) were excluded from the record after treatment. A total of 143 measurements were included in group 1, 173 in group 2.

### Data Ascertainment

The data presented were collected from two non-interventional clinical trials at the children's hospital Frankfurt. Both trials were registered at clinicaltrials.gov 2012 (Susceptibility to infections in ataxia telangiectasia; NCT02345135) and 2017 (Susceptibility to Infections, tumor risk, and liver disease in patients with ataxia telangiectasia; NCT03357978). The study was approved by the responsible ethics committee in Frankfurt (application number 504/15 and 121/12).

The study was conducted following the ethical principles of the Declaration of Helsinki, regulatory requirements and the code of Good Clinical Practice.

### Blood Analysis

AFP, GGT, AST, ALT, CRP, cholesterol, and triacylglycerol (TAG) were determined in the serum of whole blood from a total of 67 patients. We used our data base to collect the parameters retrospectively. In case of multiple blood results within <6 months, the median within that period of time was taken into consideration.

### Ultrasound

Abdominal ultrasound was performed by a pediatric radiologist (group 1 and group 2). The reports were taken from our data base.

### Neurological Assessment

All A-T patients (group 1 and group 2) were examined by a pediatric neurologist as recently described (25). Disease progression was classified according to KAS (24). Gait ataxia, standing ataxia, ataxia of the upper and lower extremity, dysarthria, intention tremor and dysdiadochokinesis were assessed. Impairment is evaluated in points from zero (nonexistent) to five (maximum deficiency) so that the maximum score is 35 points.

### Statistical Analysis

For statistical analysis GraphPad Prism 5.01 (GraphPad Software, Inc.) and R version 3.2.4 (R Foundation for Statistical Computing, Vienna, Austria) were used. Values are presented as arithmetic means with standard deviations (SDs). For comparisons between the two study groups, two-tailed Mann- Whitney- *U*-test was applied. Correlations were analyzed by Spearman's correlation coefficient. Liver enzymes (ALT, AST, GGT) were defined as primary variables. The secondary variables included AFP, CRP, cholesterol, and TAG and KAS. In addition, a multivariable linear mixed effect regression analysis was performed and normality assumptions for residuals were checked for this analysis.

*P*-values ≤ 0.05 were considered significant.

## Results

We investigated 67 patients with A-T for liver disease. Patients were divided according to age into two groups (group 1: 37 patients aged <12 years, group 2: 30 patients ≥12 years). At least two measurements of liver enzymes were performed within a minimum interval of 6 months in 56 patients (longitudinal data). The median follow-up period was 4 years (1–16 years). A total of 316 liver enzyme measurements were performed.

**Figure 1:**
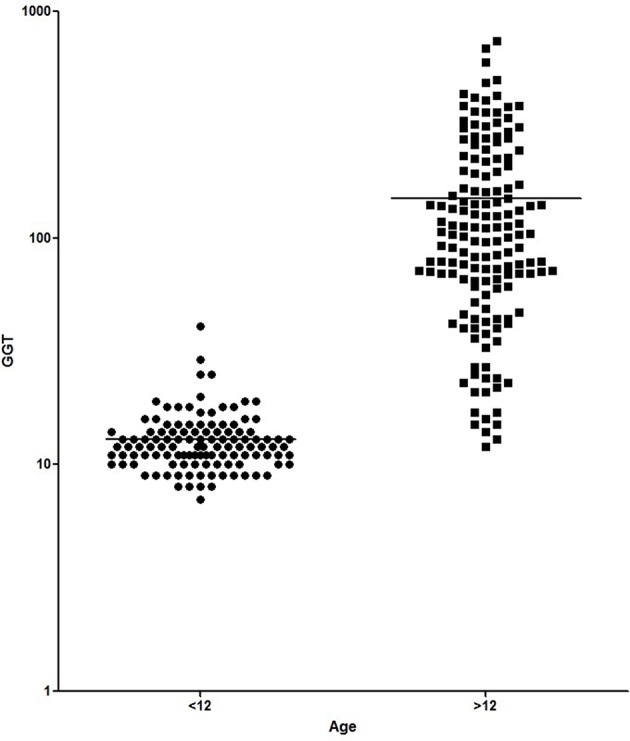
Comparison of GGT [U/L] between A-T patient <12 years of age (*n* = 127) and ≥12 years of age (*n* = 156). Graph is shown as logarithmic scale *p* < 0.0001.

**Figure 2:**
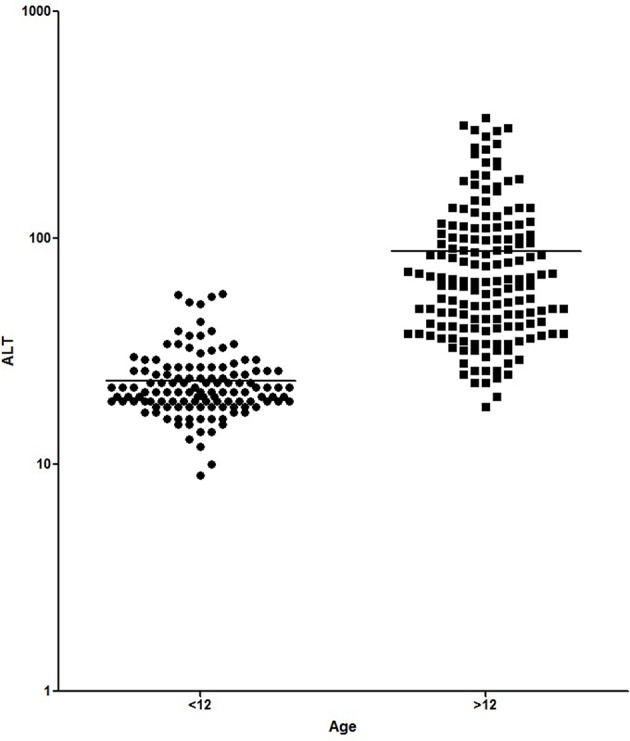
Comparison of ALT [U/L] between A-T patient <12 years of age (*n* = 138) and ≥12 years of age (*n* = 164). Graph is shown as logarithmic scale *p* < 0.0001.

**Figure 3:**
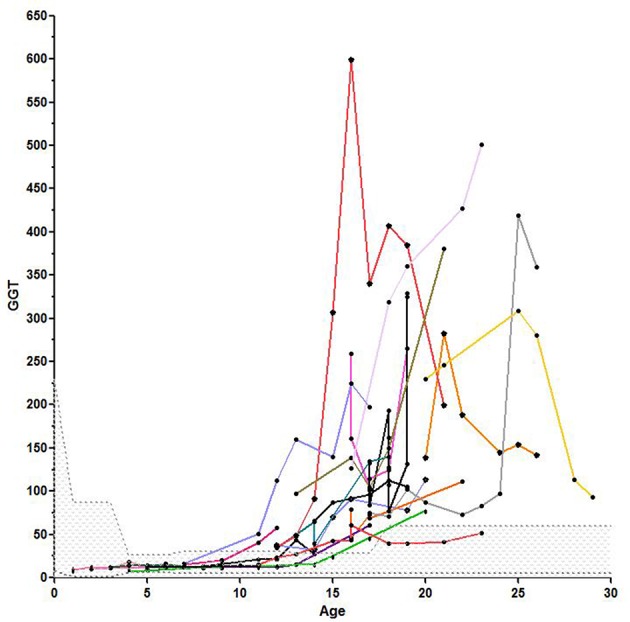
Individual course of GGT with age, the scattered area is the normal range for GGT.

**Figure 4:**
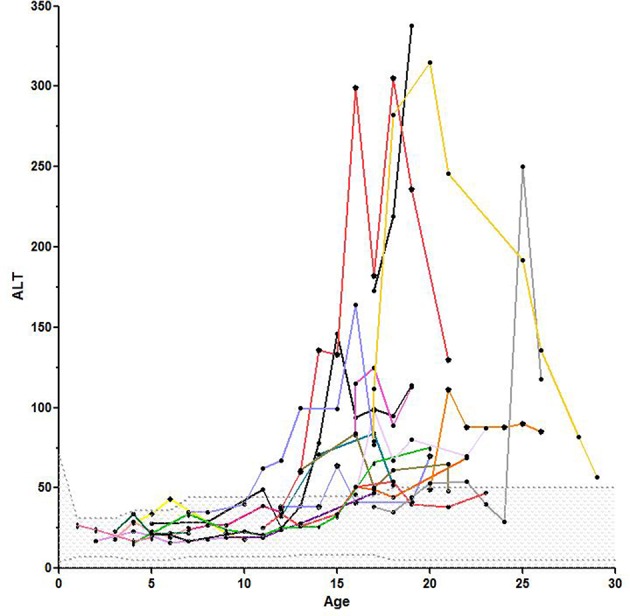
Individual course of ALT with age, the scattered area is the normal range for ALT.

Figures 1, 2 show the comparison between group 1 and 2 concerning GGT and ALT. Figures 3, 4 show individual courses of GGT and ALT with age.

Table 1 shows patient characteristics. The mean age was 6.1 years in group 1 and 18.4 years in group 2 (*p* < 0.0001). We found significantly higher levels of AFP (226.8 ± 20.87 ng/ml vs. 565.1 ± 24.3 ng/ml, *p* < 0.0001), GGT (12.98 ± 0.41 IU/L vs. 149.5 + 10.99, *p* < 0.0001) and ALT (23.52 ± 0.77 IU/L vs. 87.83 ± 5.31 IU/L, *p* < 0.0001) in older A-T patients. The analysis of AST did not show a significant difference (37.92 ± 0.75 IU/L vs. 59.72 ± 3.52 IU/L, *p* >0.05). In addition to that, CRP levels were significantly increased in the older group (0.16 ± 0.03 vs. 0.27 ± 0.04, *p* < 0.05) pointing to a chronic systemic inflammatory response, especially in older patients.

**Table 1:** Patients' characteristics.

	**Group 1:** **<12 years** ***n* = 37**	**Group 2** **≥12 years** ***n* = 30**	***P*-value**
Age^*^ [years]	6.13 ± 2.8	18.4 ± 5	<0.0001
KAS [points]	12.92 ± 0.86 n = 55	23.76 ± 0.43 n = 38	<0.0001^#^
AFP [ng/dl]	226.8 ± 20.87 n = 95	565.1 ± 24.30 n = 137	<0.0001^#^
GGT [U/l]	12.98 ± 0.41 n = 117	149.5 ± 10.99 n = 153	<0.0001^#^
ALT [U/l]	23.52 ± 0.77 n = 125	87.83 ± 5.31 n = 161	<0.0001^#^
AST [U/l]	37.92 ± 0.75 n = 126	59.72 ± 3.52 n = 163	*p* > 0.05^#^
TAG [mg/dl]	84.39 ± 8.0 n = 31	251.8 ± 22.24 n = 60	<0.0001^#^
Cholesterol [mg/dl]	176.3 ± 5.13 n = 32	212.7 ± 7.91 n = 57	<0.05^#^
CRP [mg/dl]	0.16 ± 0.03 n = 108	0.27 ± 0.04 n = 116	<0.05^#^

*Mean and SD of all quantifications are shown, n, Number of quantifications*.

* *Age at start of investigations*;

# *Group comparisons with p-values used the geometric mean of longitudinal data in each patient*.

TAG and cholesterol were significantly higher in group 2 when compared to group 1 (TAG: group 1: 84.39 ± 8.0 vs. group 2: 251.8 ± 22,24, *p* < 0.0001; cholesterol: group 1 176.3 ± 5.13; group 2: 212.7 ± 7.91, *p* < 0.05).

We could show a significant correlation between age and AFP (*r* = 0.64, *p* < 0.0001), membrane-bound GGT (*r* = 0.82, *p* < 0.0001) and cytoplasmic ALT (*r* = 0.63, *p* < 0.0001). In addition, CRP (*r* = 0.38, *p* < 0.01), TAG (*r* = 0.5, *p* < 0.01) and cholesterol (*r* = 0.46, *p* < 0.01) correlated with age. Mitochondrial AST (*r* = 0.024) did not correlate with age. Correlations are shown in Table 2.

**Table 2:** Linear Regression of AFP, GGT, ALT, AST, KAS, TAG, cholesterol, and CRP with age.

	**Parameter**	***r***	***P***
Age	AFP	0.64	<0.0001^#^
	GGT	0.82	<0.0001^#^
	ALT	0.63	<0.0001^#^
	AST	0.24	
	KAS	0.76	<0.0001^*^
	TAG	0.50	0.0026^#^
	Cholesterol	0.46	0.0069^#^
	CRP	0.38	0.0051^#^

*Correlation between age and AFP, GGT, ALT, AST, KAS, TAG, cholesterol, and CRP are shown, significant correlations could be shown for AFP, GGT, ALT, TAG, and KAS*.

# *Correlations and p-values were calculated according to the geometric mean of the longitudinal data in each patient*.

* *Correlation and p-value were calculated according to the arithmetic mean of the longitudinal data in each patient*.

**Figure 5:**
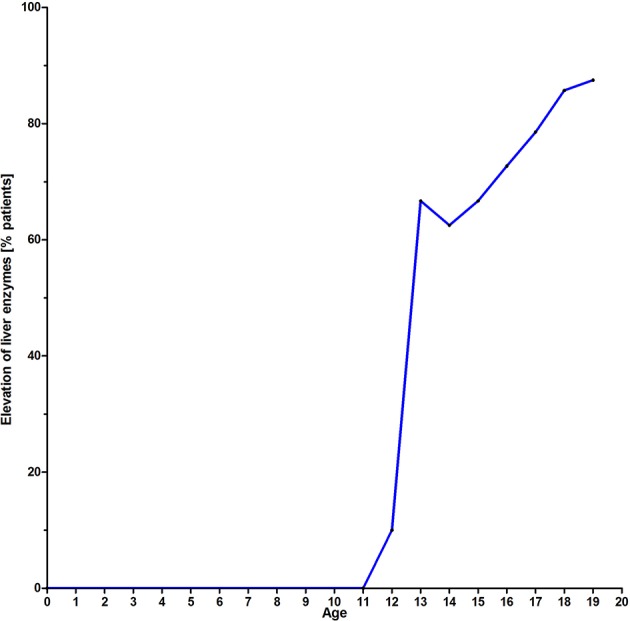
Liver enzyme elevation in percent of affected patients with age.

A significant correlation could be established between GGT and HbA1c (*r* = 0.61, *p* < 0.0001), cholesterol (*r* = 0.59, *p* < 0.0001), TAG (*r* = 0.61, *p* < 0.0001).The progressive elevation of liver enzymes is shown in Figure 5 and Table 3.

**Table 3:** Dataset on evolution of liver enzymes with age.

**Age [years]**	**0-11**	**12**	**13**	**14**	**15**	**16**	**17**	**18**	**19**	**20-38**
Measurements *n =*	143	12	11	11	8	18	16	25	10	40
Patients *n =*	37	10	9	8	6	11	14	14	8	14
Liver enzymes normal [%]	100	90 (9/10)	33.3 (3/9)	37.5 (3/8)	33.3 (2/6)	27.3 (3/11)	21.4 (3/14)	14.3 (2/14)	12.5 (1/8)	7.1 (1/14)
Liver enzymes elevated [%]	0	10 (1/10)	66.7 (6/9)	62.5 (5/8)	66.7 (4/6)	72.7 (8/11)	78.6 (11/14)	85.7 (12/14)	87.5 (7/8)	92.9 (13/14)

*The dataset shows the percentage of patients showing elevated liver enzymes with age. While before puberty (group 1, <12 years of age) hardly any patient has abnormal liver enzymes, there is an increase after the 12th year of life (group 2). Also compare to Figure 5*.

We analyzed 57 KAS in group 1 and 42 in group 2. KAS differed significantly with 12.92 ± 0.86 points vs. 23.76 ± 0.43 points, *p* < 0.0001. As expected, KAS score correlated significantly with age (*r* = 0.76, *p* < 0.0001). We could also show a significant correlation between KAS and AFP (*r* = 0.47, *p* < 0.0001) and GGT (*r* = 0.62) and ALT (*r* = 0.46). No correlation was found between KAS and AST. In addition, the multivariable linear mixed effect regression analysis showed that age and GGT were independent significant predictors for KAS. A total of 92 abdominal ultrasounds were performed. In group 1, 26 patients had undergone a total of 35 examinations. In group 2, 19 patients had performed a total of 57 examinations. Ultrasound revealed hepatic steatosis in 11/19 (57.9%) patients in group 2 and in none of the younger patients. The presence of fatty liver disease correlated significantly with age (*r* = 0.59, *p* < 0.0001). Four patients had ultrasounds analyzed in both, group 1 and 2. Twenty-one patients in both groups were never investigated with ultrasound.

One female patient aged 37 years died due to a HCC.

## Discussion

A-T is a devastating systemic disease characterized by neurodegeneration, increased risk for cancer, immunodeficiency and failure to thrive. Whereas, immunodeficiency in patients is rather static, disease progression is demonstrable at different organ systems. Liver disease was recently discovered as a new entity, and underlies progression to liver cirrhosis or HCC (7, 15).

Many of the clinical alterations in A-T are related to dysfunctional control of ROS observed in the absence of Ataxia telangiectasia mutated (*ATM*) (17, 26). In addition, early insulin resistance and a high prevalence of diabetes type 2 in older patients depend on this process (6). While gastrointestinal involvement, mainly dysphagia, poor weight gain, and failure to thrive have been well documented (1, 2, 27), hepatic and metabolic involvement in A-T is little known and understood (6, 7, 15). In this investigation we measured liver enzymes in 67 patients and had longitudinal data of 56 patients. We were able to show that GGT and ALT were significantly increased in older compared to younger patients. The mitochondrial AST was not significantly different, elevated enzyme levels of AST point to a severe hepatic damage. Elevated GGT, ALT, and AST levels were not present in group 1. In group 2 we could show the evolution of liver enzymes with age after puberty as shown in Figure 5 and Table 2. We could demonstrate a constant increase of liver enzymes after the 12th year of life up to 92.9% in the third decade of life.

For group 2, these data are in line with the recent reports of the National center in Israel and Brazil which found liver enzyme abnormalities in 43% of children and in 64% of older A-T patients (6, 15). The report from Brazil by Paulino et al. concluded that the increase in age is a risk factor for insulin resistance and liver involvement (15).

Our investigation is the first investigation of the evolution of liver disease and neurological decline longitudinally over 4 years (median, 1–16 years) in 67 patients. We could show that liver enzymes increased constantly after puberty. As expected, liver enzymes detected in the serum correlate well with the extent of liver disease. In addition to that we could demonstrate a correlation between age and liver disease (GGT, *r* = 0.82) as well as KAS (r = 0.76). A multivariate analysis confirmed the correlation of age and GGT with KAS. Of course neurodegeneration is present long before other organ systems show significant dysfunction. This concerns liver, lungs, pancreas, malignancy, and blood vessels. Our assumption is that in addition to the toxic effects on the (central) nervous system, liver involvement indicates progression of the disease as it affects one of the most regenerative and hard-wearing organs in the human body. In summary, according to the present data, we assume that patients with liver disease show a higher severity of A-T which is due to a potentially accelerated neurodegeneration and higher risk for HCC.

The available data show that GGT is the most sensitive marker and the first to increase in case of liver disease. This is logically explained by the location of GGT in the cell membrane. GGT is expressed by liver and bile tissue, but can also be found in kidney, pancreas and spleen. Therefore, it is not as specific as AST and ALT. However, most parts of serum GGT originate from the liver. Perishing hepatocytes initially set the membrane-bound GGT free. Only in severe damage the cytoplasmic ALT and lastly the mitochondrial AST will be released to plasma.

In the search for potential other risk factors of liver disease than age, we could show a significant correlation for HbA1c (*r* = 0.61, *p* < 0.0001), cholesterol (*r* = 0.59, *p* < 0.0001), TAG (*r* = 0.61, *p* < 0.0001) with GGT. CRP and IgM did not correlate.

This highlights the role of metabolic risk factors in development of liver disease. Cholesterol, elevated lipid levels and diabetes lead to fatty liver disease. We could not demonstrate a correlation between immunological findings and liver disease. Much more, our data suggest that liver disease and metabolic alterations are inherent in the *ATM* gene.

Apart from liver enzymes, we analyzed abdominal ultrasounds of the Frankfurt A-T cohort for fatty liver disease. In group 1, no patient was affected, whereas almost 60% of our older patients were affected. In addition, there was a pronounced progression of steatosis with age. One female patient died at the age of 37 due to HCC.

There have been several reports on non-alcoholic fatty liver disease (NAFLD), NASH, and liver cirrhosis as well as portal hypertension in A-T patients (7, 12, 13). NASH is histologically characterized by steatosis, swollen hepatocytes, inflammation, and fibrosis in the lobe area. With persistent necro-inflammatory inflammation and increasing liver damage, patients with NASH often develop liver fibrosis (28–30). Cirrhosis is the major clinical endpoint in NASH. Most likely A-T patients have a high risk to develop fibrotic and cirrhotic changes since ongoing inflammation with high levels of IL-6 and IL-8 have been detected before (19, 31). Chronic systemic inflammation and misled immune response as observed in granuloma and premature aging are well known features in A-T patients (2, 32, 33). Although we did not measure cytokines in our patients, mild elevated CRP levels in our older patients indicated permanent inflammatory response and acute phase reaction. The potential for NASH to progress to both, cirrhosis and later HCC, has been known for decades, but the underlying molecular mechanism in A-T patients is not elucidated so far.

Of course, metabolic imbalance, reduced glucose tolerance, and dyslipidemia cause NAFLD. In addition, oxidative stress seems to play a major role in the development and progression of liver disease. ATM can be activated by ROS directly, or by DNA double-strand breaks (34). In the absence of ATM, patients show low antioxidant capacities (26). As a result, macromolecules, lipids and DNA are exposed to permanent oxidative stress and resulting damage (17). Similar results could be shown in NASH, where oxidative stress and lipid peroxidation are increased as well (35). There is emerging evidence that the *ATM* signaling pathway plays a crucial role in the response to the accumulation of fat in the liver as demonstrated in mice (36). Hepatic fat accumulations as well as oxidative stress from free fatty acids activate *ATM*. In turn, p53 activates the pro-apoptotic gene “p53 upregulated modulator of apoptosis” (PUMA). PUMA is a major player in development of steatoapoptosis in hepatocytes (36). Since apoptosis of hepatocytes normally correlates with the severity of NASH and the stage of fibrosis, it can be assumed that steatoapoptosis is also responsible for the progression of the liver disease (1, 36, 37).

Fatigue is a well-documented phenomenon of any kind of liver disease (38). The chronic fatigue observed in older A-T patients may also result from liver involvement.

Moreover, the alterations of lipid metabolism biomarkers are suggestive of atherosclerotic risk of A-T patients and emphasize the importance of multidisciplinary care, routine monitoring of cardiovascular biomarkers and appropriate nutritional guidance (1, 7, 15). Recently, it was shown that *ATM* is also involved in metabolic and cardiovascular complications when disrupted (39, 40). In addition, *ATM* kinase deficiency aggravated left ventricular dysfunction and remodeling late after myocardial infarction in mice (41). Interestingly, mice with *ATM* haplo-deficiency had decreased vascular endothelial growth factor production and impaired angiogenesis in response to myocardial infarction, leading to accelerated heart failure (41). Indeed there is a first report which found that the atherosclerotic lipid profiling was correlated to carotid intima-media thickness in A-T patients (15). In line with this finding is an increased of cardiovascular events A-T heterozygotes reported in several studies (40, 42).

The limitations of the present study are its retrospective character, in addition to that only the KAS was used to assess neurological decline. However, the more sophisticated SARA score was only introduced in 2006 and was therefore not available for all patients.

## Conclusion

Liver disease is present in the majority of adolescents with A-T, starting after the 12th year of life. Abnormal ultrasound, elevated liver enzymes, alterations of lipid metabolism and elevated CRP suggest an atherosclerotic risk in A-T. Besides the enormous threat of progression of NASH to liver cirrhosis and HCC, liver disease may indicate a more severe disease course and aggravate neurological symptoms. Thus, prevention of liver disease is of particular importance for better well-being, better life quality and exercise endurance of our patients.

### What This Study Adds to the Field

Liver disease is present in almost all older A-T patients and begins in puberty. Structural changes, non-alcoholic fatty liver disease and fibrosis are frequent findings. Liver disease may indicate a more severe disease course and aggravate neurological symptoms.

## Data Availability Statement

The datasets analyzed in this manuscript are not publicly available. Requests to access the datasets should be directed to helena.pommerening@kgu.de.

## Ethics Statement

The studies involving human participants were reviewed and approved by Ethics committee Goethe University Clinic Frankfurt. Written informed consent to participate in this study was provided by the participants' legal guardian/next of kin.

## Author Contributions

HD helped with the study design, conducted visits, collected data, did statistical analysis, and wrote the manuscript. SZ developed the study design, conducted visits, and wrote the manuscript. SW conducted visits, collected data, and helped with the study design. RS collected data, helped with statistical analysis, and helped developing the study design. MK and MT evaluated KAS (neurological visits). UH helped collecting data and writing the manuscript. DK helped collecting data. VK helped collecting and interpreting data. EH did the statistical review.

### Conflict of Interest

The authors declare that the research was conducted in the absence of any commercial or financial relationships that could be construed as a potential conflict of interest.

## Abbreviations

A-T: Ataxia telangiectasia
GGT: Gamma-glutamyl-transferase
AST: Aspartate aminotransferase
ALT: Alanine aminotransferase
AFP: Alpha feto-protein
NASH: Non-alcoholic steatosis hepatis
HCC: Hepato-cellular carcinoma
CRP: C-reactive protein
KAS: Klockgether ataxia score
WHO: World Health Organization
TAG: Triacylglycerol
SD: Standard deviation
ROS: Reactive oxygen species
ATM: Ataxia telangiectasia mutated
NAFLD: Non-alcoholic fatty liver disease
PUMA: P53 upregulated modulator of apoptosis.
